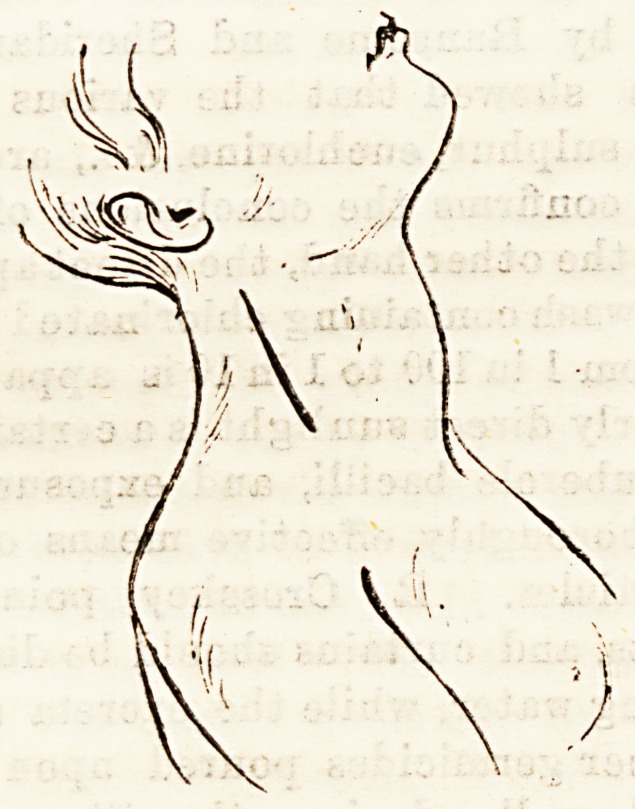# The Treatment of Pott's Disease

**Published:** 1895-07-13

**Authors:** E. Percy Paton


### Medical Progress and Hospital Clinics.

[*The Editor will be glad to receive offers of co-operation and contributions from members of the profession. All letters should be addressed to* The Editor, The Lodge, Porchester Square, London, W.]

### THE TREATMENT OF POTTS' DISEASE.

E. Percy Paton, M.D., M.S. Lond., F.R.C.S.

Potts' disease of the spine is better named tubercular disease of the vertebral column. This being the nature of the diseased process, it is essential to successful treatment that three definite aims be kept constantly in view, namely: (1) The promotion of resolution of the tubercular bone inflammation; (2) the imitation of bone destruction, and the resulting deformity caused by that destruction; (3) the promotion of ankylosis, by which means the weakened column is again able to support the weight of the body.

Secondary to these ends is the treatment of complications, such as abscess and paraplegia.

In any simple case of Potts' disease, the one method of treatment which fulfils all these aims is physiological rest, and we shall now deal with a few of the most important methods by which this rest may be obtained. First and foremost is rest in bed. This is absolutely necessary in all cases which are at all acute, as a preliminary to the use of any form of apparatus, until the acute symptoms have passed off.

In disease of the dorsal or lumbar regions either the dorsal or the prone position may be employed. The latter has the advantage of placing the diseased region highest, and so prevents passive congestion of the part. It tends to overcome or prevent the formation of the angular deformity, and also it sometimes relieves pain better than the dorsal position. It is best carried out in the case of children (and it is little applicable to the treatment of adults) on a sloping board, at the upper end of which is a horizontal part, on which may be placed the child's toys, &c. This board looks not unlike a much enlarged writing-slab. On it the child may be kept all day, being moved with the greatest care to the dorsal position in a cot at night. In either position it will have to be prevented from indulging its desire to sit up by wearing a brace consisting of a band across the chest, having two loops, one at either end, through each of which an arm is passed; another band is then passed through the two armlets, behind the child's back, and round the mattress of the cot.

In cervical disease the supine position is a necessity, as here the head must be controlled. This is best done by placing a large, heavy brick, well wrapped up, on each side of and close to the patient's head. It need hardly be said that during the time of confinement to bed the most scrupulous cleanliness, absolute dryness, and careful attention to all the details of nursing must be paid, to prevent the formation of pressure sores. Should the angular deformity of the spine be so marked as to be pressed upon severely when the child lies down, it must have the pressure taken off it by carefully padding rings of tow, &c., made like small lifebuoys, which distribute the pressure on to the surrounding parts.

If while this treatment is being carried out the patient can be moved out into the open air just as it lies whenever the weather is the least bit suitable, and is at the same fed with nutritious food and given cod-liver oil, &c., while a certain amount of exercise is obtained by diligent massage of the limbs every day, no better method can be devised. It must be persevered in for at least six months, even in early and slight cases, and then only gradually given up, and with great watchfulness.

There are, however, few patients or patients' friends who will submit to such a treatment for very lengthened periods, and fewer still who can afford the constant attention it requires; and certainly unless fresh air, as well as recumbency can be obtained, it is better, as soon as the acute symptoms have quite passed off, to maintain rest of the part by some form of apparatus which enables the patient either to get about, or be easily moved about. The number of kinds of apparatus is legion, but only a few of the more generally serviceable will be referred to here.

First comes Sayre's plaster jacket, the application of which may be briefly described as follows. The patient is clothed in a tightly fitting woven vest. If a child he may then be held up by the shoulders, so that only part of the body weight is supported by the toes. Plaster bandages are then put on steadily, and not too tightly, extending from the armpit to just below the iliac crests, while at intervals vertically placed strips of perforated tin are put in to strengthen the case. It may be wise in some cases to put in a small pad over the epigastrium, which may be taken out when the case is hard and allows room for expansion when food is taken. If the patient be an adult he is best supported by a pulley, which is attached to a soft collar fixed round his neck, the cord of which he himself may pull if desired. This extension does all that can be done as to separating the diseased bones, and, it is to be feared, it is but little. If the curve be very prominent it may be protected from pressure by stitching a pad to the undervest on each side of the projection. The iliac crests, from which the jacket takes its points of support below, may be protected in the same way. If the disease be in the cervical region an upright bar of iron called a jury mast may be fixed into the jacket whilst it is being put on; this is then bent over, and the head supported by suspension from it; the result is usually not very good, and other forms of treatment for these cases is preferable.

Two great advantages are at once evident in the plaster jacket, namely, the ease with which it can be applied with material ready to hand, and its cheapness, It has, however, also two great disadvantages, namely, its weight and the fact that although it can, it is true, be cut down the front and taken off and then reapplied; this so soon hopelessly damages the case that it can hardly be said a plaster jacket can be taken on and off.

Other forms of jacket are those made of leather or patent poroplastic felt. The latter does very well, but requires a large oven in which to soften the felt so that it can be moulded on to the patient. It has the advantage that the portion which comes in contact with the deformity can be left soft, so that it does not rub against the skin in the situation. But both leather and poroplastic really require an instrument maker to fit them properly. To either of them a jury-mast can be attached.

In cervical cases it is, however, much better if leather or poroplastic be used, to have with the former material a collar forming the upper part of the jacket, or with the latter a headpiece moulded so as to take in the whole head and neck as well as the chest, but leaving out the face, as recommended by Mr. Walsham. These jackets are lined with chamois leather, and fastened with laces and hook-eyelets or straps up the front.

With any of these jackets the patient can get about with safety; but of course great care must be taken, and only very gentle exercise allowed, especially at first.

There are two other forms of rigid apparatus which must be mentioned; these are the steel brace and the double Thomas' hip splint. The former requires a good deal of technical knowledge for its proper application, so it will be merely mentioned here. The double Thomas, on the other hand, is very easily made by any blacksmith under supervision, and is easily applied, as in an ordinary hip case, and by extension of the two bars upwards to a ring of iron carefully padded with leather, on which will lie the occiput, kept in place by a webbing strap across the forehead, it can be made useful for even cervical cases.

Though this apparatus will not allow the patient to get about himself, it allows him to be freely moved without danger; and in children, by prolonging the leg bars beyond the feet, they can be even stood up against the wall on these iron legs with perfect safety.

In order to have a permanent record as to the increase or diminution of the deformity (which, of course, is some index as to the progress of the case), it is well before any apparatus is applied to take a moulding of the curve in the following way: A piece of composition gas piping is obtained and moulded to the shape of the spinal column; it is then put on a large sheet of cardboard and the curves marked with a pencil, and cut out with scissors. The cut edge of the card can now be placed against the back, and, if necessary, further parings made with scissors until it exactly fits. Subsequently, similar tracings may be made and compared with that first taken, and any diminution or increase of curve easily noted.

The next question to be answered is, How long must this absolute rest of the part be maintained? The shortest time is six months, and this only in very slight and early cases. Nine or twelve months, or even longer still, are often, indeed, commonly necessary. The indications for cessation of the treatment are complete absence of pain, at rest and on movement; diminished if not complete absence of rigidity of the muscles in the affected area of the column, and fixity of the deformity, showing that the inflammatory trouble has stopped, and the parts have become ankylosed.

We must now turn to the treatment of the two chief complications of spinal caries, namely, abscess and paraplegia.

The former is a very common complication, just as it is in tubercular caries in any other situation, and it must be met in the same way, that is, by removal of the pus, at the same time removing, as far as practicable, the caseating granulations lining the abscess cavity, along with any loose fragments of bone.

The most common form of abscess is that which, commencing under the anterior common ligament of the vertebral column, spreads into the sheath of the psoas muscle. It may be opened in two situations either at the anterior superior iliac spine, or in the lumbar region.

The former is the easier operation. The abdominal wall is cut through, till the peritoneum is reached, by an incision from two to three inches long, its centre being placed one inch from the iliac spine, and parallel to Poupart's ligament. The peritoneum is then pushed towards the middle line, if the abscess has not already done this. The sac wall is then seized with pressure forceps to prevent it slipping out of sight when the pus escapes, a small opening is now made with the knife, and enlarged with dressing forceps, and the cavity explored by the finger to see if any dead or carious bone can be reached. The abscess is then thoroughly washed out with boiled water, and its walls cleared from caseous granulations by systematic scrubbing with sponges held on long forceps, or by means of Barker's flushing curette. It is after this again washed out, and this treatment repeated until the washings come away only blood-stained. The wound is then stitched up, and a tube left in for twenty-four hours to allow of the escape of the large quantity of serum which exudes in consequence of the large raw surface produced by the scraping. The quantity of exudation is also diminished by well-applied pressure. After removal of the tube the wound should not require dressing for a week, when it should be found healed.

As this anterior incision does not permit the upper part of the cavity to be reached, a lumber opening has been advised by Treves, placed between the last rib and iliac crest along the outer border of the erector spinal, that is to say from two and three-quarter to three inches from the vertebral spines. The next landmark is the top of the second, or better the third, lumbar transverse process, and by keeping close to this, and separating the quadratus lumborum, a small opening into the psoas may be made, which can be enlarged by blunt instruments without danger to the lumbar arteries or nerves. The great advantage of this incision is, that the cavity is more directly reached; but better still the bodies of the vertebræ can be explored with the finger and thoroughly treated if much diseased.

For either of these operations it is necessary that the strictest asepsis be maintained, otherwise they are fraught with no little danger.

It need hardly be said that not infrequently a re-collection of pus occurs in the abscess cavity, which only partially becomes obliterated; but there is no difficulty in repeating the process a second, or even a third, time, and if rigid treatment by rest, good food, fresh air, &c., be at the same time pursued, satisfactory results may in most cases be expected.

Should the cavity become septic, the freest possible drainage, with frequent, if not constant, irrigation, present the best prospect of a successful issue.

A reference must now be made to the form of abscess known as post-pharyngeal, which occurs in connection with cervical disease. This may, of course, be easily opened through the mouth, but this method is unsatisfactory, in that asepsis cannot be maintained, nor can the seat of disease be dealt with. It is, therefore, better to reach the pus in most cases by an incision placed along the posterior border of the sterno-mastoid. This muscle is then retracted inwards, and the pus reached by dissecting with blunt instruments, keeping close to the front of the vertebræ, and behind the sheath of the carotid vessels. Care must be taken to avoid injuring either the phrenic or the sympathetic nerves.

Paraplegia may be treated on two different principles, namely, actively or passively. The latter method is suited to most cases—at any rate, at first. It consists in merely waiting for the results of rest and good nursing, and in a large number or cases the paralysis clears up as the disease becomes quiescent, the pressure on the cord being due not so much to bone deformity as to inflammatory products thrown out into the canal outside the dura mater. If, however, after prolonged expectant treatment, the palsy increases or remains quite stationary, or if the sphincters become affected or the patient appears to be going progressively downhill, it becomes not only proper, but imperative, for the surgeon to interfere, and to explore the spinal canal by removal of two or three laminæ opposite the most prominent deformity. By this means it may be possible to let out some fluid, to dissect off some tubercular granulation tissue, or even to remove some sequestra which may be pressing upon the cord, in which case, even after prolonged paralysis, recovery may take place. Too sanguine a view, however, must not be taken of the prospects following operation in these cases. It need hardly be said that in all cases in which paraplegia occurs the formation of pressure sores must be prevented by the use of a water-bed, absolute cleanliness and dryness, and the most scrupulous attention to every other detail of nursing.

## Figures and Tables

**Figure f1:**
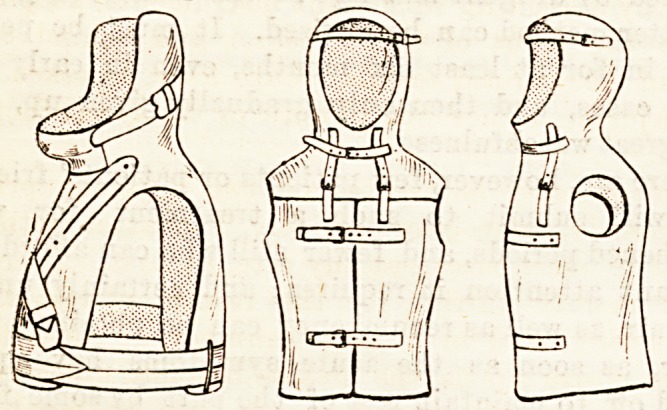


**Figure f2:**
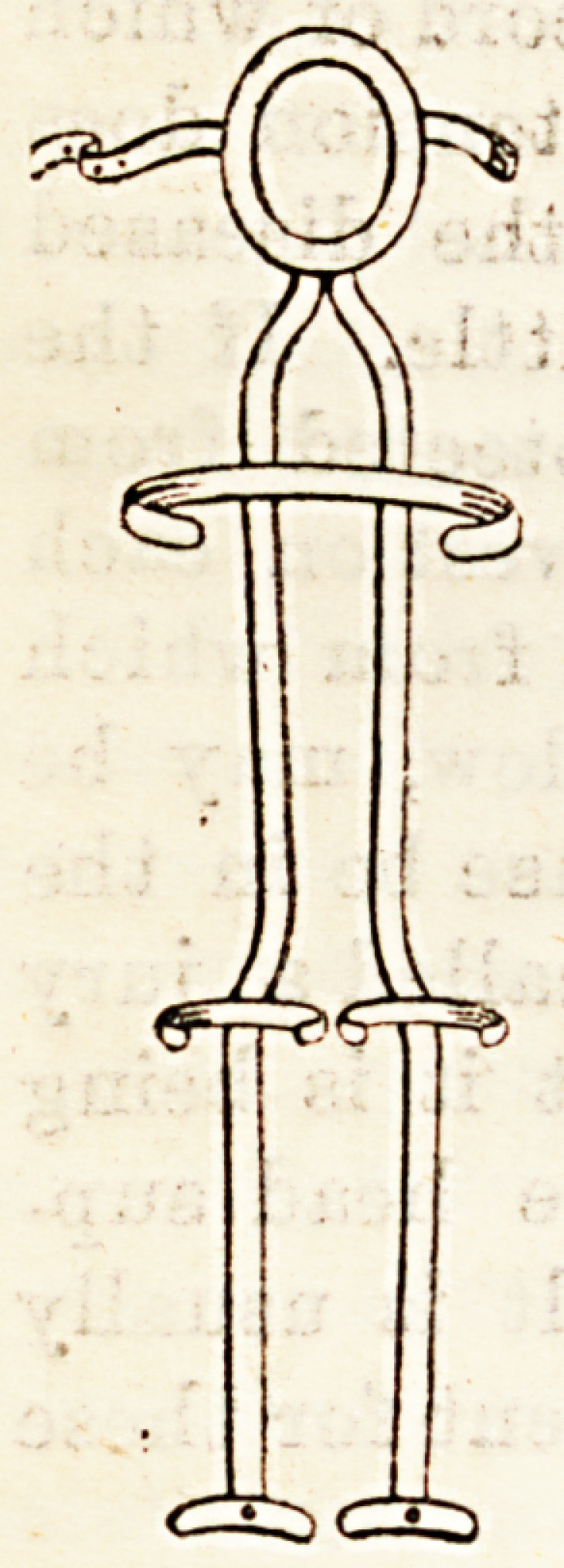


**Figure f3:**
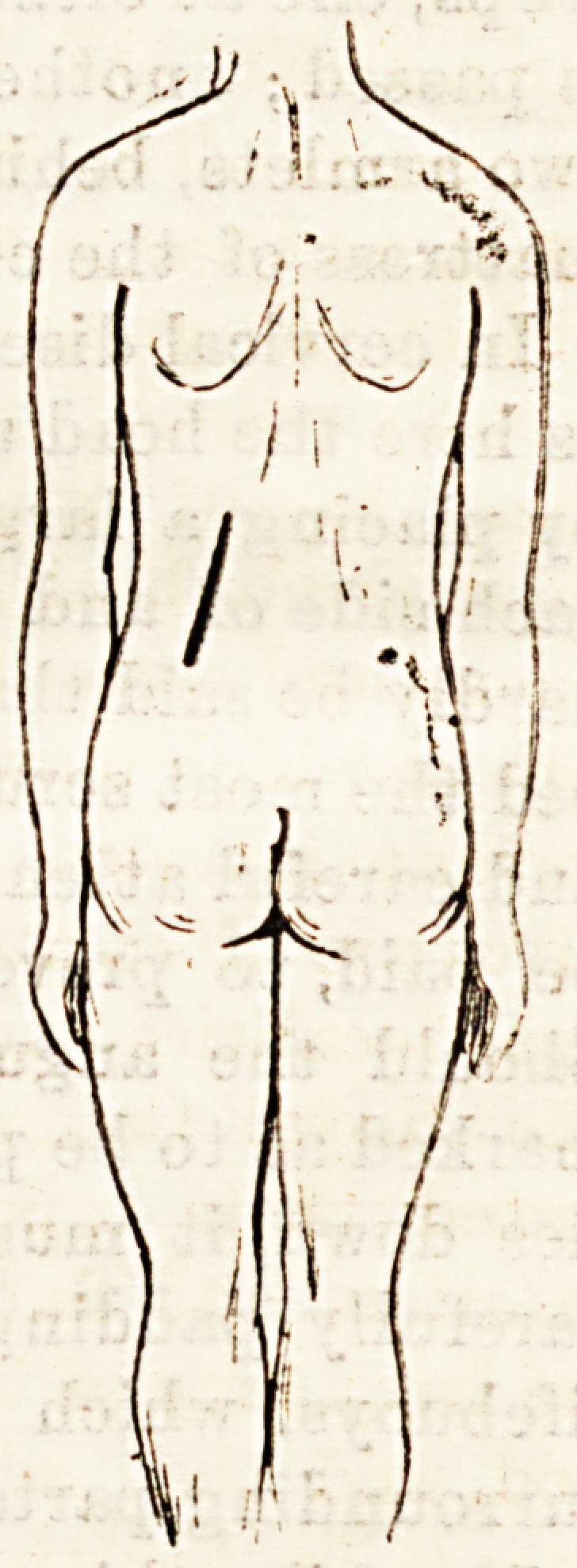


**Figure f4:**